# Draft genome sequence of *Pantoea ananatis* strain MHSD5 isolated from surface sterilized leaves of medicinal plant, *Pellaea calomelanos* obtained in South Africa

**DOI:** 10.1016/j.dib.2018.06.039

**Published:** 2018-06-22

**Authors:** Siphiwe Godfrey Mahlangu, Mahloro Hope Serepa-Dlamini

**Affiliations:** Department of Biotechnology and Food Technology, Faculty of Science, University of Johannesburg, Doornfontein Campus, PO Box 17011 Doornfontein 2028, Johannesburg, South Africa

## Abstract

*Pantoea ananatis* strain MHSD5 is a bacterial endophyte isolated from the surface sterilized leaves of *Pellaea calomelanos,* which is a medicinal plant obtained in Limpopo province of South Africa. We present here the draft genome sequence and annotation of *P. ananatis* strain MHSD5. The genome assembly was 4.6 Mb in size with an N50 of 550,557 bp. A total of 4,350 putative protein coding sequence genes were predicted with PGAAP. This is the first draft genome of a bacterial endophyte symbiotically associated with *P. calomelanos*. This Whole Genome Shotgun project has been deposited at DDBJ/ENA/GenBank under the accession PUEK00000000. The version described in this paper is version PUEK01000000.

## Specifications Table

**Table:** 

Subject area	Biology
More specific subject area	Plant-microbe interaction, Bacteriology, Genomics, Bioinformatics
Type of data	Table, figure
How data was acquired	Genome sequencing: Illumina MiSeq at Inqaba Biotechnological Company, Pretoria, South Africa,
	De novo sequence assembly: Web-based Galaxy Unicycler version 0.4.1.1, Bioinformatics approaches: NCBI Prokaryotic Genome Automatic Annotation Pipeline (PGAAP), Rapid Annotation using Subsystem Technology server (RAST).
Data format	Analysed
Experimental factors	Genomic sequencing, assembly and annotation
Experimental features	The whole genome of *Pantoea ananatis* strain MHSD5 was sequenced on Illumina MiSeq sequencing platform. Read quality was assessed using Galaxy platform-FastQC version 0.69. *De novo* assembly of the reads were performed by Galaxy web platform-Unicycler version 0.4.1.1, and quality of assembly was assessed with Quast Genome assembly Quality version 4.6.3 (https://usegalaxy.org).
Data source location	*P. ananatis* strain MHSD5 was isolated from surface sterilized leaves of *P. calomelanos* obtained from Botlokwa, Limpopo Province, South Africa (23°29׳34.8"S 29°42׳11.2"E).
Data accessibility	Genome assembly,annotation and analysis of data are found in this article and the raw data together with NCBI PGAAP annotation were deposited at the NCBI repository:
	https://www.ncbi.nlm.nih.gov/bioproject/PRJNA434382,
	Bioproject ID: 434382, BioSample: SAMN08555277
	This Whole Genome Shotgun project has been deposited at DDBJ/ENA/GenBank under the accession PUEK00000000. (http://www.ncbi.nlm.nih.gov/nuccore/PUEK00000000)
	The genome annotation performed at RAST server are also given in this article.

## Value of the data

• The first draft genome of bacterial endophyte symbiotically associated with *Pellaea calomelanos*.
• The whole genomic data provided information on genetic components of *Pantoea ananatis* strain MHSD5 involved in endophytic life style.
• *Pantoea* genus comprise species associated with various hosts, thus the genome information will advance genome comparison of various *Pantoea* species and further provide insights into the biology and evolution of the genus.

## 1 Data

The genus *Pantoea* has diverse species, which have been isolated from several environments such as aquatic and terrestrial environments [1]. *Pantoea* genus currently has twenty described species which have associations with humans, plants, insects, and animals [1]. Association of *Pantoea* species with various hosts can be parasitic, mutualistic or commensal [1], [2]. The genus consists of yellow-pigmented, gram-negative, rod-shaped bacteria in the Enterobacteriaceae family. Although most species have been reported to have pathogenic associations with humans, animals and plants [3], [4], few have been reported to be symbiotically associated with plants [5]. As plant endophytes, *Pantoea* bacteria have been reported to promote plant growth via a variety of mechanisms and produce bioactive compounds with antibiotic activities [6], [7], [8], [9].

Since *Pantoea* species have associations with different hosts in different environments, there is need for understanding genetic factors which allow this group of bacteria to successfully colonize various hosts. In addition, the availability of various genomes of *Pantoea* genus will promote whole genome comparison within this group and further our understanding of genetic factors that contribute to *Pantoea* species thriving in different environments and thus delineating their biology and evolution.

We recently isolated *Pantoea ananatis* strain MHSD5 from surface sterilized leaves of *Pellaea calomelanos*, a medicinal plant utilized for treatment of chest colds, asthma, headaches, head colds as well as mouth and nasal ulcers [10]. Initial identification of the bacterium was confirmed by sequencing of its 16S rRNA gene, which was deposited in GenBank with accession number MF613651. This is the first study to isolate, identify and report on the draft whole genome sequence of bacterial endophyte associated with *P. calomelanos.*

DNA sequencing was performed with Illumina MiSeq platform which generated 250 Mb data of reads. The genome assembly of *P. ananatis* strain MHSD5 produced 39 contigs, with N50 of 550,557 base pairs (bp), the largest contig with 1,441,770 bp. The genome of *P. ananatis* strain MHSD5 was 4,650,216 bp, with GC% content of 54.16%, which falls within the genome sizes (4.5–6.3 Mb) and GC% content (52–55%) ranges of some of the sequenced *Pantoea* species. Genome annotation was performed with Prokaryotic Genome Automatic Annotation Pipeline (PGAAP) and Rapid Annotation using Subsystem Technology server (RAST) [11], [12]. *P. ananatis* strain MHSD5 has 4,437 genes, among the identified genes 4,350 are protein coding sequence genes (CDS) and 119 are pseudogenes. The genome also has 3 rRNAs with five operons (5S, 16S and 23S) and 70 tRNAs genes.

**Fig. 1:**
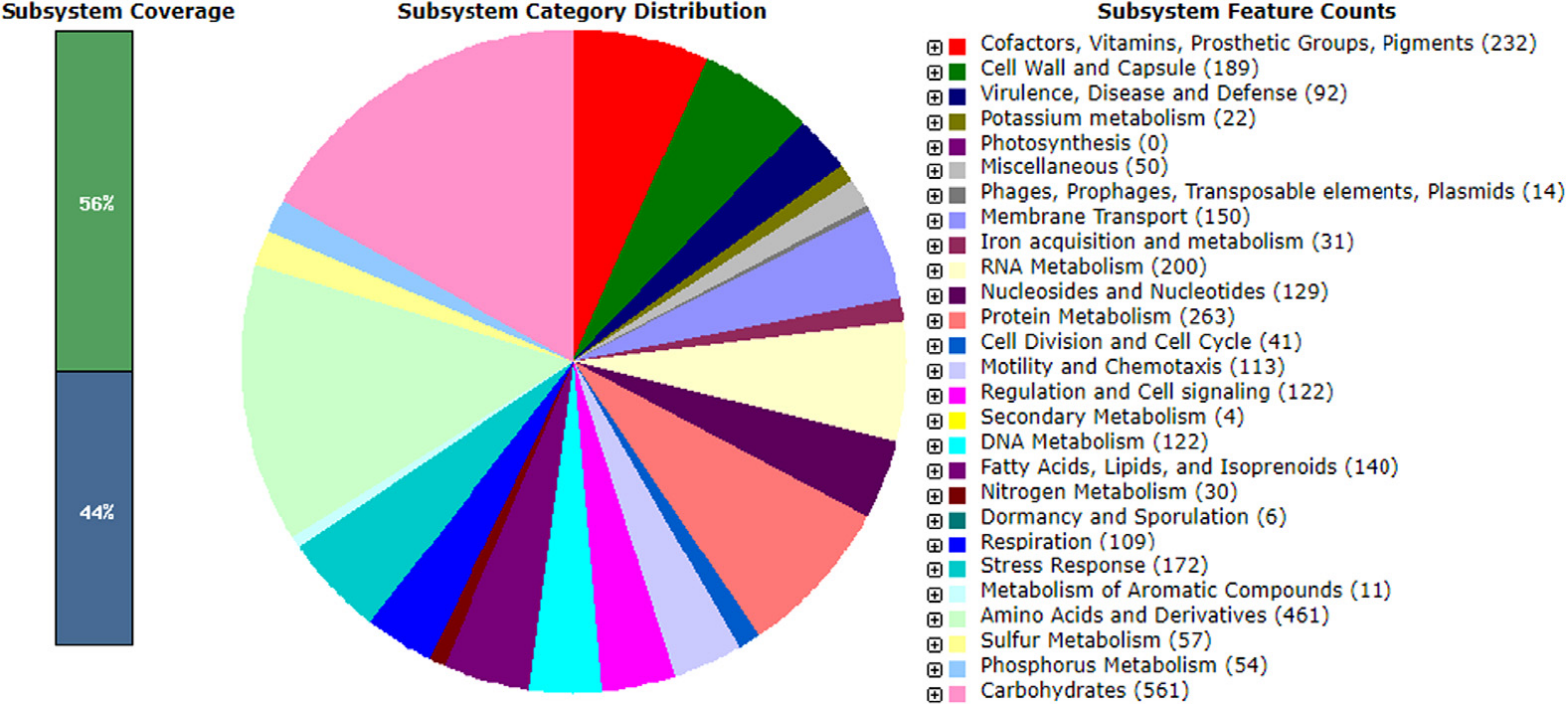
The subsystem distribution of *Pantoea ananatis* strain MHSD5 generated from RAST annotation server.

**Fig. 2:**
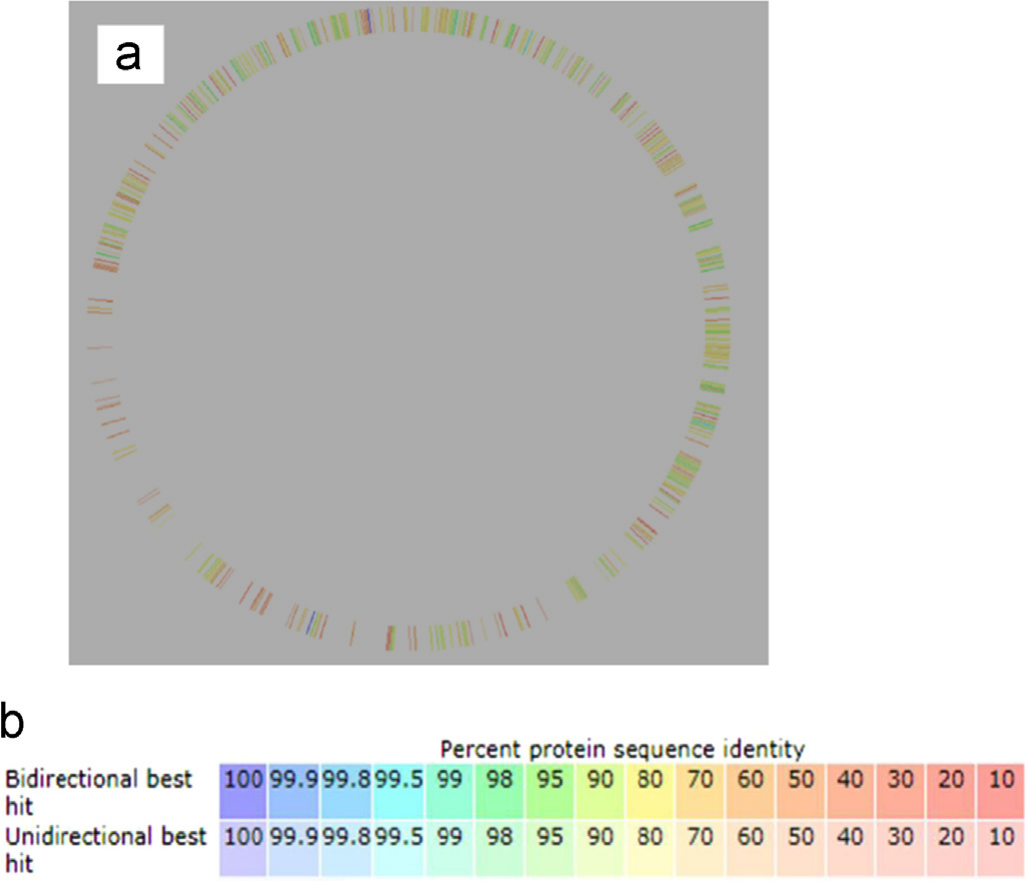
(a) *Pantoea ananatis* strain MHSD5 genome compared to *P. stewartii* DC283, with the latter used as reference genome, (b) colour co-ordination similarity of the genome comparison in percentages. Bidirectional best hit refers to both forward and reverse hits.

PGAAP and RAST annotation pipelines resulted in minor differences (Table 1). We registered the annotation results from PGAAP on GenBank. The RAST Subsystem Information (Fig. 1), shows the subsystem feature counts, with 92 genes involved in virulence, disease and defence; and carbohydrates having the most number of genes of 561 and 4 genes of secondary metabolism which are the lowest number of genes. Through the RAST sequence based comparison tool [13], we compared the assembled genomes of *P. ananatis* strain MHSD5 and *Pantoea stewartii* DC283 [14], using the latter as a reference genome as shown in Fig. 2(a) and (b). Although there were genome gaps on *P. ananatis* strain MHSD5 (Fig. 2(a)), it showed 70–99% similarity in protein sequences of both reverse and forward (bidirectional hits) to *Pantoea stewartii* DC283.

**Table 1:** Outcome comparison of *Pantoea ananatis* strain MHSD5 genome annotation using PGAAP and RAST.

**Genomic feature**	a**PGAAP**	**RAST**
Total number of genes	4437	4397
Protein coding genes	4350	4324
Number of RNAs	87	73
Contigs	39	39
N50	550,557 bp	550,557 bp
GC%	54.16%	54.2%

a Only the PGAAP results were registered with GenBank.

Genomics is crucial in identifying genes that are important for the bacterial endophyte to successfully penetrate, colonize and have symbiotic association with its plant host. In accordance to the life style of endophytes, we identified genes involved in nitrogen fixation, production of plant hormone indole acetic acid (IAA), production of antibiotics, toxins as well as toxin-antitoxin genes. Similar genes have been previously reported in other endophytic bacteria such as *Azoarcus* sp. strain BH72 as well as *Gluconacetobacter diazotrophicus Pal5* [15], [16]. Some of the identified toxin and antitoxin genes are similar to *Enterobacter* sp. 638, a plant growth promoting endophyte which was fully sequenced and annotated [17].

## 2 Experimental design, materials and methods

### 2.1 Genome extraction and sequencing

*P. ananatis* strain MHSD5 was isolated from surface sterilized leaves of *P. calomelanos* obtained from Botlokwa, Limpopo Province, South Africa (23°29׳34.8"S 29°42׳11.2"E). Whole genomic DNA of *Pantoea ananatis* strain MHSD5 was extracted from nutrient agar pure colonies using Quick-DNA™ ZR fungal/bacterial DNA MiniPrep kit (Zymo Research, catalogue #D6005) according to manufacturers’ protocol. The extracted DNA was cleaned with ZR fungal/bacterial DNA clean and concentrator-5 (catalogue #D4003). The whole genome sequence was performed at a commercial service provider Inqaba Biotechnological Company PTY (LTD.), Pretoria, South Africa. Briefly, genomic DNA sample was fragmented using an ultrasonication approach (Covaris). The DNA library was prepared using the NEBNext Ultra™ II DNA Library Prep Kit for Illumina (New England BioLabs Inc.) according to the manufacturers’ protocol. The resulting fragments were size selected, end repaired and Illumina specific adapter sequences were ligated to each fragment. Following quantification, the samples were individually indexed and a second size selection step was performed (AMPure XP Bead-based), and sequenced on Illumina MiSeq platform, using a MiSeq v3 (600 cycle) kit. 250 Mb of data (2 × 300 bp long paired end reads) was produced.

### 2.2 Genome quality assessment, de novo assembly and annotation

Quality assessment of raw reads was performed by FastQC version 0.69. The reads were *de novo* assembled using Unicycler version 0.4.1.1 and the assembly was assessed by Quast version 4.6.3. The assembly generated 39 contigs with an N50 of 550,557 bp and 53× coverage. All the pre-annotation analysis were performed on Galaxy web platform (https://usegalaxy.org) [18]. The NCBI Prokaryotic Genome Automatic Annotation Pipeline (PGAAP) was used to perform initial annotation, which was followed by Rapid Annotation using Subsystem Technology (RAST).
